# Clustering of cognitive phenotypes identifies susceptible and resilient offspring in a rat model of maternal immune activation and early-life stress

**DOI:** 10.1016/j.bbih.2022.100514

**Published:** 2022-09-20

**Authors:** Jarred M. Lorusso, Rebecca M. Woods, Francesca McEwan, Jocelyn D. Glazier, Joanna C. Neill, Michael Harte, Reinmar Hager

**Affiliations:** a Division of Evolution, Infection, and Genomics, School of Biological Sciences, Manchester Academic Health Science Centre, Faculty of Biology, Medicine & Health, University of Manchester, Manchester, M13 9PL, United Kingdom; b Division of Pharmacy & Optometry, School of Health Sciences, Manchester Academic Health Science Centre, Faculty of Biology, Medicine & Health, University of Manchester, Manchester, M13 9PL, United Kingdom

**Keywords:** Maternal immune activation, Early life stress, Infection, Animal model, Two-hit, MIA, Poly(I:C), Schizophrenia, Clustering, Cognition

## Abstract

Schizophrenia and other neurodevelopmental disorders often have very heterogeneous symptoms, especially regarding cognition: while some individuals may exhibit deficient cognition, others are relatively unaffected. Studies using developmental animal models often ignore phenotypic heterogeneity in favour of traditional treatment/control comparisons. This may result in resilient or unaffected individuals masking the effects of susceptible individuals if grouped together. Here, we used maternal immune activation and limited bedding and nesting, respectively, as a two-hit neurodevelopmental model for schizophrenia. Both factors reduced cognitive function in a novel object recognition (NOR) task. While we found treatment group effects on cognitive phenotypes, behavioural clustering identified three subpopulations exposed to either insult: those exhibiting ‘typical’ cognitive performance on the NOR, an intermediate phenotype, or a marked deficit. These clusters included offspring from each treatment group, although both intermediate and marked deficit clusters were composed primarily of offspring from treated groups. Clustering allowed stratification within treatment groups into ‘susceptible’ and ‘resilient’ individuals, while also identifying conserved phenotypes across treatment groups. Using unbiased cluster analyses in preclinical models can better characterize phenotypes and enables a better understanding of both face and construct validity of phenotypic heterogeneity. The use of unbiased clustering techniques may help identify potential markers associated with individual susceptibility and resilience in neurodevelopmental disorder models.

## Highlights

• Maternal immune activation and early life stress affect recognition memory.
• New groups were identified using unbiased clustering based on task performance.
• These clusters reflected high-, low-, and intermediary recognition memory.
• Treatment offspring were distributed across all three unbiased clusters.
• These findings suggest some offspring will be resilient to developmental insults.

## 1 Introduction

Neurodevelopmental disorders (NDDs) such as schizophrenia present as complex psychiatric conditions associated with significant cognitive deficits, and positive and negative symptoms. Cognitive deficits in particular can appear heterogeneous between individuals with shared diagnoses, despite having been diagnosed based on the same criteria (Van Rheenen et al., 2017)**.** Clinical studies have noted large heterogeneity in the cognitive profile of patients with schizophrenia (Gilbert et al., 2014), suggesting that diagnosis as the sole means of patient classification in research ignores the inter-individual variability within each group (Van Rheenen et al., 2017). Recent studies stratifying patient populations on the basis of phenotype rather than diagnosis (or experimental manipulation) have been able to utilize this variability to identify symptom-specific patient subgroups, better characterizing their individual symptomatic profile (Clementz et al., 2016; Gilbert et al., 2014; Van Rheenen et al., 2017). For instance, the Bipolar-Schizophrenia Network for Intermediary Phenotypes Study (B–SNIPS) identified three patient clusters based on cognitive performance and sensorimotor reactivity, composed of individuals with bipolar disorder with psychosis, schizophrenia, or schizoaffective disorder. These groups did not reflect diagnostic differences, instead sharing similar symptomatic profiles (Clementz et al., 2016). In schizophrenia, cognitive deficits are seen in most patients, who can be stratified into subgroups based on the degree to which they exhibit general or domain-specific deficits (Gilbert et al., 2014). These subgroups may represent distinct neurobiological subgroups as suggested by the B–SNIPS and supported by subgroup-specific responses to treatment (Clementz et al., 2016; Gilbert et al., 2014).

As in the clinical context, pre-clinical models often overemphasize the importance of the treatment or manipulation rather than the output, i.e. the measured behaviour. For example, neurodevelopmental models focus predominantly on the manipulation of the prenatal environment, as this period shows the greatest sensitivity to risk factors of neuropsychiatric disorders (Howes and McCutcheon, 2017). In particular, exposure to infection *in utero* has been linked to an up to five-fold increase in the relative risk of NDDs, specifically schizophrenia (Brown and Derkits, 2010). This scenario can be modelled in pregnant rodent dams by exposure to an immunogen such as polyinosinic:polycytidylic acid (poly (I:C); a viral mimetic immunostiumulant) resulting in maternal immune activation (MIA) (Murray et al., 2019). Exposure to maternal infection can result in neurodevelopmental insults through fetal neuroinflammation (Lipina et al., 2013) or altered placental function (Kowash, 2020). Additionally, these prenatal insults may prime specific responses to postnatal stressors such as material deprivation (Omer et al., 2014) or adult maltreatment (Arseneault et al., 2011), which increase the relative risk of psychotic symptoms (Arseneault et al., 2011; Howes and McCutcheon, 2017). One method of modelling these stressors is through limited bedding and nesting (LBN), in which reduced bedding material is provided to the litter. LBN may impart its effects through altering maternal behaviour. This is seen through an increase in fragmented nursing behaviour, such as licking and grooming, as well as impaired nesting behaviour, (Gallo et al., 2019; Walker et al., 2017), illustrating an appropriate analogue for human maltreatment (Arseneault et al., 2011). This model has been shown to result in phenotypes related to schizophrenia and other NDDs such as cognitive deficits and reduced sociability (Walker et al., 2017). Models of both types of risk factors can be combined into preclinical, two-hit neurodevelopmental models, enabling studies of both main and interaction effects between stressors (Giovanoli et al., 2013).

While these models allow for greater control over covariates, they often find large variability in the observed phenotypes between individuals within a treatment group. One approach to appreciate this variability in preclinical models is through stratification, similar to that in clinical research. Once unbiased groups are identified based on phenotypes (e.g. ‘exposed-and-resilient’ or ‘exposed-and-susceptible’), biomarker analyses can then identify specific *in vivo* markers of susceptibility. While inter-individual variability has been described by previous research (Van Rheenen et al., 2017), only recently have studies begun to identify potential sub-populations. For example, Mueller et al. (2021) identified a subpopulation of MIA-exposed mice based on a high-inflammatory profile that differed socially from low-inflammation MIA offspring and control animals. The use of clustering and subject stratification to provide better identification of subtype-specific dysfunctions can thus promote personalised tailored therapeutics.

Our study aims to provide support for clustering based on behavioural outputs obtained in a rat model of MIA, with significant advances over previous stratification approaches in animal models. We modelled two environmental risk factors for schizophrenia: MIA (Murray et al., 2019) and limited bedding and nesting during lactation (LBN; Walker et al., 2017). Following exposure to one or both insults, adolescent offspring were tested on their visual recognition memory. Clustering allows for a ‘diagnosis’ in preclinical models akin to practices in clinic, ultimately capturing resilience in manipulated groups and idiopathic occurrence of phenotypes in control groups.

## 2 Methods

### 2.1 Animals

Wistar rat dams (*N* = 18; Charles River, UK) and their offspring (*n* = 67, *n=* 1–2M, 1–3F per litter) were used in our study. Wistar rats were selected as we have shown in our previous research that this strain, compared with others, shows low within-group variability across phenotypes, in particular in their cytokine response to immune stimulation (Murray et al., 2019). There were four treatment groups; vehicle, vehicle with stress, MIA, and MIA with stress (see section 2.3 for details). Litters were reduced to *n=10* of equal sex number (5M, 5F) to ensure equal resource access and maternal attention. Following litter reduction, dams and litters were allocated to the LBN stress condition or control rearing (*N=*4*–*5 dams per treatment group, *n=* 7–10M + 8–9F offspring per treatment group, *N* = 10, *n=*67 total). Animals were housed in the University of Manchester Biological Services Facility in accordance with the Animals (Scientific Procedure) Act of 1986 under project licence authority, with local ethical approval. Nulliparous females (mean weight: 257.4 ± 6.7g) were acclimatized to the housing conditions for at least one week prior to mating. All animals were maintained at a temperature of 21–23 °C (55–60% relative humidity), on a 12h:12h light:dark schedule (lights on at 0730h). All animals were housed in individually ventilated cages with split-level environmental enrichment (GR1800 Double-Decker Cage, Tecniplast, UK), with *ad libitum* access to standard rat chow (Special Diet Services, UK) and water. Offspring were weaned on postnatal day (PD) 28 and then housed with littermates in cages of up to five females or up to three males.

### 2.2 Maternal immune activation (MIA)

Virgin female rats were time-mated, with gestational day (GD) 1 defined as the day a vaginal plug was found. From GD1-GD14, dams were pair-housed. On GD15 between 0900h and 1100h, dams were injected intraperitoneally with either 10 mg/kg bodyweight poly (I:C) (low molecular weight, Invivogen, France) or injected with an equivalent volume of vehicle control (endotoxin-free 0.9% NaCl, Invivogen). Dams were monitored following injection for any adverse reaction (none were noted). The first day pups were seen was designated as PD0.

### 2.3 Limited bedding and nesting (LBN)

Following litter reduction on PD1, dams and litters allocated to the LBN stress condition were placed into a clean cage with an elevated metal platform approximately 2.5 cm above reduced bedding material (1.5 cm of woodchips in LBN). Control litters had access to 2.5 cm of woodchip bedding. Both conditions were given equal nesting material (70–85g Sizzlenest), though LBN litters could not make an adequate nest (Walker et al., 2017). Litters were returned to typical nesting conditions on PD10.

### 2.4 Novel object recognition (NOR)

We focused on cognitive outputs as these show high levels of inter-individual variability in clinical settings, and because of their prior use as a clustering factor in clinical research (Clementz et al., 2016; Gilbert et al., 2014). Offspring cognition was tested through the Novel Object Recognition (NOR) task, reflecting visual learning and memory, one cognitive subdomain affected in schizophrenia (Nuechterlein et al., 2004). NOR deficits are seen in multiple models for schizophrenia in rodents, including dopamine agonism, glutamate antagonism, neonatal ventral hippocampal lesions, and MIA (Lyon et al., 2012). Further, NOR is recommended as an analogue to human visual learning and memory tasks due to its ease of testing as well as its involvement of regions affected in patients (such as the frontal cortex) or those involved in the cognitive subdomains affected in the disorder such as the perirhinal cortex (Young et al., 2009). Offspring were first habituated to the plexiglass testing arena for two days before testing. On the testing day, offspring were placed back into the testing arena which now contained two identical objects (two opaque glass bottles or two Diet Coke cans). This was the acquisition phase. Rats were allowed to explore the objects for 3 min, after which offspring were removed and placed into their home cage for 2 min, when the items were removed, and arenas cleaned with 70% ethanol. Following cleaning, a previous item was returned and a novel item was placed in the arena. The animal was then placed back in the centre of the arena and allowed to explore either object for a further 3 min (testing phase). Items were counterbalanced to account for any side preference within the arena. Exploratory time in the acquisition phase was first analysed to ensure no side preference.

Exploratory time in the testing phase was analysed as Discrimination Index (DI), defined as:

$$DI=\;\frac{Novel\;Item\;Exploration\;\left(s\right)\;–Familiar\;Item\;Exploration\;\left(s\right)}{Novel\;Item\;Exploration\;\left(s\right)+Familiar\;Item\;Exploration\;\left(s\right)}$$

where a score of +1 represents an exclusive novelty preference, while a score of −1 represents an exclusive familiarity preference. DI was analysed both for group differences, and as a one-sample *t*-test against zero, indicating no object preference.

### 2.5 Statistics

Statistical analysis was performed using SPSS v.25.0 (IBM) and visualized with GraphPad Prism v9.0 (Graphpad, USA). Power analyses were run on comparable cognitive task data from our laboratory (d = 1.082) and comparable research (D = 1.446, Mattei et al., 2014) and identified *N* = 10 dams as sufficiently powered for up to two-way interactions at α = 0.05 and a power of 0.8.

Exploration times for items in the acquisition phase were analysed by repeated measures ANOVA and DI was analysed using a General Linear Mixed Model (GLMM) with prenatal treatment, postnatal stress, and sex as fixed factors while accounting for dam as a random factor. When post-hoc analyses were needed, GLMMs were used in order to account for the effect of the shared prenatal environment within litters. A two-step unbiased clustering analysis was used with no pre-determined number of clusters to avoid any experimenter bias. DI was used as the sole input in determining clusters. Cluster quality was analysed as part of the two-step algorithm in SPSS via silhouette indices rated on a scale of −1 to +1. A greater score indicates greater distance between clusters and a tighter fit of datapoints within clusters, with scores above 0.5 suggesting ‘reasonable’ or ‘strong’ structure (Kaufman and Rousseeuw, 2009).

## 3 Results

### 3.1 Novel object recognition validation

To ensure that baseline side preference within the apparatus did not affect the subsequent item preference, we analysed the acquisition phase exploration preference. In the acquisition phase, animals exhibited no preference toward either side of the box irrespective of sex (F_1,61_ = 0.034, p = 0.855), LBN (F_1,61_ = 0.011, p = 0.918) or MIA (F_1,61_ = 2.688, p = 0.106). Total exploratory time in the testing phase (e.g. one novel object and one familiar object) was reduced by LBN (F_1,61_ = 7.380, p = 0.009) but not MIA (F_1,61_ = 1.593, p = 0.212) or sex (F_1,61_ = 0.021, p = 0.885).

### 3.2 Novel object recognition test

One-sample t-tests identified that control (VEH) offspring (t_15_ = 7.394, p < 0.001) and vehicle-stressed (VEH+) offspring (t_18_ = 3.246, p = 0.005) exhibited a significant novelty preference, illustrating baseline capacity for item recognition. Both MIA-unstressed (MIA, t_15_ = 0.262, p = 0.797) and MIA-stressed (MIA+, t_18_ = 1.726, p = 0.103) offspring did not. Novelty preference in the testing phase was affected by MIA (F_1,61_ = 6.816, p = 0.011) and an interaction between MIA and LBN (F_1,61_ = 4.086, p = 0.048, data not shown). DI was reduced by exposure to MIA (F_1,60_ = 8.746, p = 0.004). Sex (F_1,60_ = 0.023, p = 0.881) and LBN (F_1,60_ = 0.191, p = 0.664) did not have a main effect on DI. As sex did not show a significant effect, analyses include combined male and female data. Post-hoc analyses indicate that, when DI was compared against VEH offspring, VEH+ (F_1,32_ = 4.547, p = 0.041), and MIA (F_1,29_ = 14.808, p = 0.001) differed significantly, while MIA + did not (F_1,5.897_ = 4.881, p = 0.070).

### 3.3 Clustering analysis

**Fig. 1:**
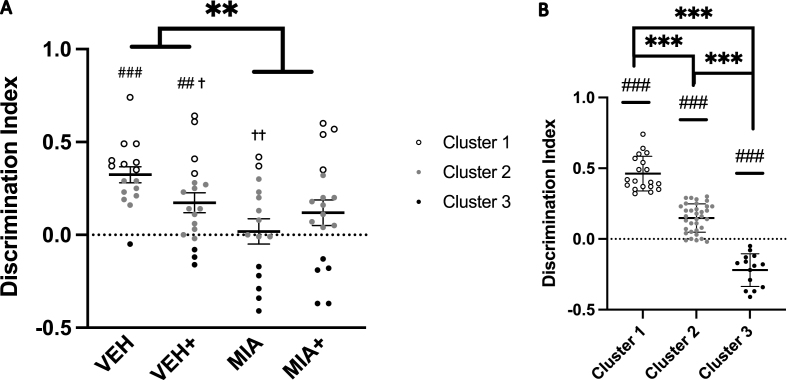
**Offspring cognition in the Novel Objection Recognition Task**. A) Prenatal poly (I:C) reduced novel object preference in adolescence. LBN reduced preference in vehicle animals but had no effect in poly (I:C)-exposed offspring. N = 4–5/group, n = 15–18/group. B) Clustering based on performance resulted in three groups of varying size that significantly differed from one another and illustrated an object preference. N = 10–19/cluster, n = 14–34/cluster ** = p < 0.01 compared across treatment conditions. *** = p < 0.001 compared across treatment conditions. ## = p < 0.01 compared against 0.0 (no object preference). ### = p < 0.001 compared against 0.0 (no object preference).† = p < 0.05 compared to VEH, †† = p < 0.01 compared to VEH. + = Limited bedding and nesting material. Data presented as individual scores with group mean ± SEM.

**Fig. 2:**
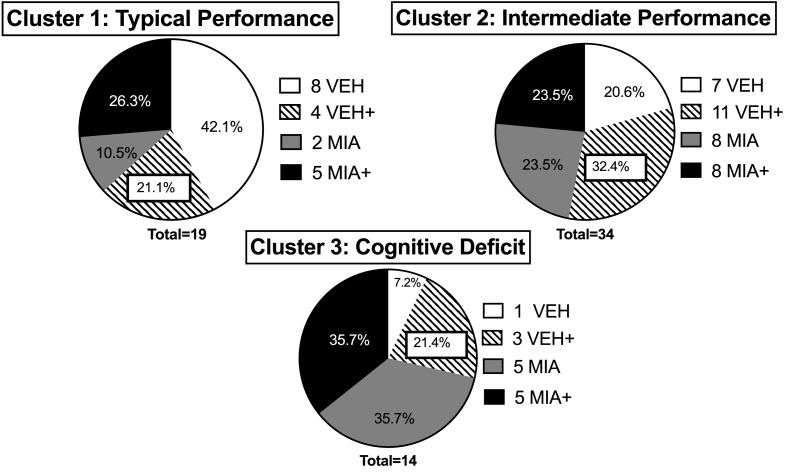
**Cluster Proportion by Treatment**. The composition of each cluster is presented with the number of offspring from each treatment indicated. VEH offspring compose the largest proportion of cluster 1, which decreases as performance decreases and deficit becomes more pronounced. This is in contrast to MIA and MIA + offspring, which make up a progressively larger proportion of the impaired cluster. VEH + offspring are predominantly represented in clusters 2 and 3.

We identified three clusters (Fig. 1B), reflecting a silhouette index of cohesion and differentiation of 0.6, rated as “good” in SPSS or a ‘reasonable’ structure (Kaufman and Rousseeuw, 2009). Cluster 3 (Fig. 1B), reflecting the most severe cognitive deficit, included only a single control animal (Figs. 1A and 2), whereas control offspring made up the largest proportion of cluster 1 (Fig. 2), the least-affected cluster. The DI differed significantly between clusters (F_2,64_ = 156.980, p < 0.001, Fig. 1B). Additionally, all three clusters differed from zero in one-sample t-tests, with a novel object preference for cluster 1 and 2 (t (18) = 16.552, p < 0.001; t (33) = 8.701, p < 0.001) contrasting with a familiar object preference for cluster 3 (t (13) = -7.140, p < 0.001). While MIA did not affect distribution into these clusters overall (χ^2^ = 3.991, p = 0.136), it did affect distribution in offspring who were not exposed to stress (χ^2^ = 6.308, p = 0.043). MIA offspring were less populous in the typically-performing cluster and more populous in the most-affected cluster when compared to VEH offspring.

## 4 Discussion

Our study demonstrates that behavioural clustering in rat models of NDDs can stratify individuals into groups based on their potential susceptibility to the insults. Importantly, the group membership of these clusters differs from the simple binary grouping commonly used (treatment vs vehicle), and is in line with unbiased comparisons of schizophrenia-spectrum disorders using cognition in the clinic (e.g. Van Rheenen et al., 2017), and using behaviour or inflammation in mouse models (Mueller et al., 2021). Interestingly, MIA did affect distribution to these clusters but only in the absence of postnatal stress.

Poly (I:C)-induced MIA and the environmental stress of LBN during early development showed distinct effects on offspring cognition. MIA-exposed offspring significantly differed from VEH offspring in object recognition and exhibited no novelty preference, irrespective of stress. VEH + offspring exhibited a reduced novelty preference compared to VEH offspring. The influence of LBN concurs with previous studies (Moussa-Tooks et al., 2020) and the interaction suggests no joint effect of both insults on cognition. Sex had no significant impact on cognition, suggesting similar developmental insults in visual recognition memory across the sexes.

We were able to identify three clusters of typical, intermediate, and deficient cognition, differing in the relative proportions of treatment groups and significantly different from one another. These results indicate a high degree of translational validity similar to that seen in epidemiological research. There was a five-fold greater number of MIA-exposed offspring composing cluster 3 (deficient cognition) compared to vehicle-unstressed offspring (Fig. 2). Irrespective of postnatal stress, MIA-exposed offspring constituted more than 70% of the most affected cluster (#3). Conversely, there were approximately two times and four times more VEH offspring in the least-affected cluster (#1) relative to either stress condition or to MIA on its own (Fig. 2). Considering the most-affected cluster (#3) to represent a ‘diagnostic threshold,’ then these proportions closely resemble those in the clinical literature (Arseneault et al., 2011; Brown and Dertkis, 2010). This aligns appropriately with the elevated risk for schizophrenia or psychotic symptom development seen in epidemiological studies resulting from both prenatal infection (Brown and Derkits, 2010) and postnatal socioeconomic deprivation (Arseneault et al., 2011). Therefore, cluster membership appears to mimic the relative risks reported in the literature (Fig. 2). Prenatal treatment significantly affects cluster membership in the absence of postnatal stress, such that allocation to the clusters is different between MIA and VEH offspring. Interestingly, this is not the case when both stressed and unstressed offspring are combined, nor across prenatal treatments following stress. This suggests that LBN does impact cognitive outcome, but affects MIA and VEH offspring similarly.

It is worth discussing the use of outbred Wistar rats in the current research, and the potential for genetic variation as a source of increased phenotypic variability among groups, especially in the context of reproducibility across laboratories (Crabbe et al., 1999). While some behavioural traits do support the notion of reduced variability in inbred strains compared to outbred, this appears to be less significant than previously believed (Tuttle et al., 2018). Further, inbred strains used for MIA still result in marked variability within treatment groups (Mueller et al., 2021) similar to what we found in our study. This suggests that the variability in these models may be due to individual fetal-placental responses, rather than baseline genetic differences due to strain type, and should be investigated in future research. These differences may be underpinned by dissociable epigenetic modifications on genes critical in neurodevelopment which have been seen following MIA (Woods et al., 2021).

Clustering as shown here allows us to better observe the efficacy of the model and to identify mediators of the relationship between model exposure and subsequent phenotype (or lack thereof). As clinical research begins to more readily use comparable analyses to identify patient subpopulations (Clementz et al., 2016; Gilbert et al., 2014), it is critical for preclinical and basic research to do the same (e.g. Mueller et al., 2021). Unbiased clustering can highlight resilient or susceptible subpopulations in models of environmental risk factors like those modelled here. While identifying statistical outliers within a treatment group may suggest resiliency to the insult, those outliers are still compared to their particular treatment group; ultimately this analysis is still oriented around ‘treatment.’ However, using clustering, differences may be identified that could explain the rates of non-response across manipulations (e.g. neuroanatomical or transcriptional differences). By analysing data in this way, better construct validity is achieved in models for NDDs that will also advance our understanding of the mechanisms that drive the occurrence of disorder in some individuals but not in others.

## Declaration of competing interest

The authors of this manuscript report no conflicts of interest.

## Data Availability

Data will be made available on request.
